# Assessing the impact of outdoor farming, farm size, and farm density on highly pathogenic avian influenza epidemics: A modelling study in the Netherlands

**DOI:** 10.1016/j.onehlt.2026.101424

**Published:** 2026-04-22

**Authors:** Thanicha Chanchaidechachai, Arjan Stegeman, Francisca C. Velkers, Jose L. Gonzales, Egil A.J. Fischer

**Affiliations:** a Department of Farm Animal Health, Faculty of Veterinary Medicine, Utrecht University, Utrecht, the Netherlands; b Center of Excellence in Data Innovation for Sustainable Livestock Production, Department of Veterinary Medicine, Faculty of Veterinary Science, Chulalongkorn University, Bangkok, Thailand; c Department of Epidemiology, Bioinformatics and Animal Models, Wageningen Bioveterinary Research, Lelystad, the Netherlands

**Keywords:** Avian influenza, Outdoor, Poultry farms, Disease model, Between-farm transmission, Exposure

## Abstract

Poultry farming in the Netherlands is moving toward more extensive systems to promote animal welfare, a shift that has led to the rising number of organic and free-range farms, which provide poultry with outdoor access, lower maximum stocking densities, and limited flock sizes. However, the effect of these changes on the risk of infectious diseases for poultry remains unclear. In this study, we assessed the effects of these changes on the risk of highly pathogenic avian influenza (HPAI) introduction from the environment into poultry farms and HPAI spread between farms. We focused on the changes in farm production system in broiler and layer farms, which are the majority of the poultry population in the Netherlands. The increasing number of outdoor farms increased the introduction risk of HPAI and HPAI epidemic size (number of affected farms), with outdoor layer farms contributing more substantially to the risk than outdoor broiler farms due to the higher susceptibility of layer farms. Spatial clustering of outdoor farms sharply increased the epidemic size. Farm size reduction and decreasing the number of farms reduced the epidemic sizes. Although outdoor farming practices can promote better animal welfare, they potentially increase risk of HPAI introduction and spread. However, this risk can be mitigated by preventing clusters of outdoor farms, reducing farm size, or decreasing the overall number of farms.

## Highlights

• More outdoor farms increase the risk of HPAI introduction and epidemics.
• Reducing farm size and the total number of farms mitigates these risks.
• Clustering of outdoor farms increases risk and should be prevented.

## 1 Introduction

Highly pathogenic avian influenza (HPAI) is an important zoonotic disease with serious consequences to the poultry sector and a potential threat to public health. In the Netherlands, a large HPAI (H7N7) epidemic occurred in 2003 that resulted in depopulation of 30 million birds [1], and the virus was confirmed in 86 people directly involved in handling infected poultry [2]. That epidemic was controlled and the virus eradicated with no new HPAI virus outbreak reported until 2014, when a HPAI epidemic caused by H5N8 virus was observed. Since then, HPAI (H5Nx) epidemics have continued to occur, with 129 outbreaks reported in poultry farms between 2014 and 2022 [3]. For these H5Nx HPAI viruses, wild birds act as the main reservoir [4], while spillover infections have also been reported in wild mammals [5], highlighting the importance of one health perspective and the potential zoonotic risk.

The Netherlands is seeing a shift toward more extensive poultry farming systems, largely to improve animal welfare [6], [7]. This has led to a rise in extensive production systems, including organic and free-range farms [6], which provide poultry with outdoor access, lower maximum stocking densities, and limited flock sizes [8], [9]. In the Netherlands, the number of poultry in organic and free-range system has increased from 5 million in 2005 to 9.2 million in 2020 [10]. Changes in farming practices, farm size and farm density inevitably affect the risk for HPAI in poultry farms. For example, previous studies have shown that outdoor farming increased the risk of HPAI epidemic due to the higher risk of indirect contact with wild birds [11], [12], while the reduction of farm size and the farm density decreases HPAI transmission rate between farms [13]. The overall impact of these changes in farming practices on the risk of HPAI introduction and spread in poultry is still unclear. Thus, this research aims to explore the impact of an increasing number of outdoor poultry farms, reduced farm size, and a lower number of farms on the risk of HPAI introduction and HPAI epidemic.

## 2 Materials and methods

### 2.1 Data

Data of Dutch poultry farms in 2024, including geographical coordinates, poultry type, farming system and farm size, were obtained from the Rijksdienst voor Ondernemend Nederland (RVO, The Netherlands) (Table 1). The distribution maps of poultry types are shown in supplementary S5. The farm production system was classified into indoor and outdoor, with outdoor production systems consisting of organic farms and free-range farms (Vrije uitloop in Dutch). Environmental variables for estimating the risk of HPAI introduction, including water sources and land use, were retrieved from the PDOK platform (PDOK, Publieke Dienstverlening Op de Kaart, the Netherlands) [14], [15].

**Table 1:** Summary of the number of farms and the number of animals for the different poultry production systems.

Poultry production systems^1^	Number of farms (% of farms from total poultry farms)	Percentage of indoor/outdoor of poultry type^2^	Farm size
			Median (Q1;Q3)
Indoor layers	465 (24.0)	52.5%	43,963 (21,883; 86,810)
Outdoor layers	421 (21.7)	47.5%	21,403 (11,549; 39,045)
Indoor broilers	666 (34.4)	93.5%	40,000 (21,120; 71,020)
Outdoor broilers	46 (2.4)	6.5%	8028 (4362; 18,000)
Layer breeders	47 (2.4)	100%	26,717 (16,036; 40,067)
Broiler breeders	226 (11.7)	100%	25,695 (16,118; 39,094)
Meat ducks	22 (1.1)	100%	15,126 (7732; 32,350)
Duck breeders	6 (0.3)	100%	5474 (3937; 7425)
Meat turkeys	28 (1.4)	100%	17,652 (9383; 24,026)
Mixed farm^3^	9 (0.5)	100%	23,798 (12,025; 53,286)

1 Poultry type (layers, broilers, ducks, turkeys) and production systems (indoor/outdoor).

2 Note that all turkeys and breeders in the Netherlands are kept indoors.

3 Mixed farm are the farms with more than one poultry types in the same farm.

### 2.2 HPAI introduction from the environment into poultry farms

The probability of HPAI introduction into a poultry farm was calculated based on odds ratios of poultry production system, geographical location and environmental factors [16]. The odds ratios are shown in Supplementary S4. We used the relative risk of low pathogenic avian influenza (LPAI) introduction for outdoor farming [17] to estimate probability of introduction in outdoor farms, due to the lack of reported odds ratios for indoor and outdoor farming. Since introductions were rare, the odds ratio and relative risk are approximately equal. The risk of HPAI introduction from environment into the susceptible farm *j* is calculated as follows:

(1) $$p_{j}=\frac{1}{1+e^{-\left(\beta_{0}+\beta_{j}X_{j}\right)}}\;$$

Where $\beta_{0}$ is the intercept of the logistic regression (−3.466). The $\beta_{j}$ is the vector of regression coefficients for the environmental factors (equivalent to the log of the odds ratio), and the $X_{j}$ is the vector of explanatory variables for farm *j.* The risk is interpreted as the probability of the farm getting infected with HPAI from the environment at least once within 9 years, assuming the association between variables and HPAI introduction remains the same as the period of 2014–2022. Area risk is calculated as the average of farm risks within a 10 km^2^ radius. This distance was chosen to align with the regulatory surveillance zone for HPAI.

### 2.3 HPAI epidemic: between-farm transmission model

A spatially explicit SIR model was used to simulate HPAI epidemic [18]. In this model, each farm is treated as an epidemiological unit and can be in one of three states: susceptible, infectious, and removed states. The simulation begins with all farms in the susceptible state, except for a single randomly selected index farm, which is initialized as infectious. The duration of the infectious period of an infected farm is determined using a stochastic within-farm SEIR model. Technical details of the within-farm are provided in the supplementary S1.

The transmission event in which a susceptible farm is infected, is simulated using a Sellke construction [19]. Each susceptible farm has a stochastic tolerance to infection. A susceptible farm is infected at the moment that the cumulative force of infection by all infectious farms acting on it exceeds the tolerance. When tolerance to infection is drawn from an exponential distribution with a mean of 1, the epidemic corresponds to the SIR model [18].

The force of infection acting on susceptible farm *j* at time *t* can be described as follows:

(2) $$\lambda_{j}\left(t\right)=\sum_{\mathrm{i\epsilon infectious}}k\left(r_{\mathrm{ij}}\right)f^{c}\left(n_{i},n_{j}\right)f^{s}\left(S_{j}\right)\;$$

The function $k\left(r_{\mathrm{ij}}\right)$ is the transmission kernel function, which describes the transmission rate between two farms depending on the Euclidean distance between farms ($r_{\mathrm{ij}}$) [20]:

(3) $$k\left(r_{\mathrm{ij}}\right)=\frac{k_{0}}{1+{\left(\frac{r_{\mathrm{ij}}}{r_{0}}\right)}^{\alpha }}$$

The parameter $k_{0}$ represents the transmission rate at distance of zero. The parameter $r_{0}$ represents the distance at which the transmission rate decreases to half. The parameter α determines the shape of the transmission kernel.

The function $f^{c}\left(n_{i},n_{j}\right)$ is the farm size function that determines the relation between the force of infection and the number of animals in infectious farm *i* and susceptible farm *j.*

(4) $$f^{c}\left(n_{i},n_{j}\right)=\left(1-\exp\left(-\frac{n_{j}}{\theta }\right)\right)\left(1-\exp\left(-\frac{n_{i}}{\theta }\right)\right)$$

Where $n_{j}$ and $n_{j}$ is the number of animals in the susceptible farm *j* and infectious farm *i*, respectively. The θ is a constant scaling the relation with farm size.

Independent of the size of the farm, we determine $f^{s}\left(S_{j}\right)$, the susceptibility of farm *j,* based on the composition of poultry type, as follows:

(5) $$f^{s}\left(S_{j}\right)=\frac{\sum_{k\in \mathrm{animal types}}\left(\sigma_{k}S_{j}\left[k\right]\right)}{n_{j}}$$

Where $\sigma_{k}$ is the relative susceptibility of poultry type k. The $S_{j}\left[k\right]$ is the number of poultry type k in susceptible farm *j*. The $n_{j}$ is the total number of poultry in the susceptible farm *j*.

The simulation was run for 1000 iteration for each scenario. For each run, the number of infected farms, epidemic duration and the percentage of major epidemic (i.e., the number of infected farms ≥10) were recorded and summarized. The model was programmed in R version 4.3.1 [21] and the code is available in the GitHub repository: https://github.com/AnnThanicha/HPAI-Between-farm-transmission.

#### 2.3.1 Parameterization

Parameters for susceptibility of farms and transmission were taken from the report by Hagenaars et al. (2023) [13], which were estimated using HPAI epidemic data in the Netherlands from 2003 to 2018. However, these parameters did not distinguish between the susceptibility of indoor and outdoor farming systems. According to Bouwstra et al. (2017) [17], the relative risk of LPAI introduction is 6.3 times higher in outdoor layer farms compared to indoor layer farms. Based on this, we assumed that the susceptibility of outdoor layer and broiler farms is 6.3 times higher than that of their indoor counterparts. Consequently, we calibrated the $k_{0}$ parameter from the transmission kernel so that simulations under the baseline scenario, using the adjusted susceptibility values, would produce results consistent with those obtained using the original parameters.

The calibration was done by identifying the new $k_{0}$ parameter that produced reproduction numbers for individual farms that were closest to those obtained using the original parameters. The calibrated value of $k_{0}$ was 0.000574. Outbreak simulations using the calibrated $k_{0}$ parameter produced outbreak outcomes comparable to those simulated using the original parameters, indicating that the calibrated parameters preserved the transmission dynamics of the original parameters. Details of the calibration are provided in Supplementary S3. All model parameters are described in Table 2.

**Table 2:** The parameters regarding between-farm transmission model of highly pathogenic avian influenza.

Parameter	Value	Source
Index case	Randomly select from farms in high-density area, which are farms with the 5th percentile highest point density within a 1 km radius.	Authors
Transmission kernel function	$k_{0}$ = 5.74 × 10^−4^ day^−1^ $r_{0}$ = 2.5 α = 2.2	Calibrated from [13]^1^
Relative susceptibility of poultry production system	$\;\sigma_{j}=\left\{\begin{array}{c} 1,\mathrm{if}\;j=\mathrm{indoor layer}\;\\ 6.3,\mathrm{if}\;j={\mathrm{outdoor layer}}^{2}\\ 1,\mathrm{if}\;j=\mathrm{layer breeder}\\ \;0.134,\mathrm{if}\;j=\mathrm{indoor broiler}\;\\ \;0.84,\mathrm{if}\;j={\mathrm{outdoor broiler}}^{2}\;\\ \;1,\mathrm{if}\;j=\mathrm{broiler breeder}\\ 3,\mathrm{if}\;j=\mathrm{turkey}\\ 0.377,\mathrm{if}\;j=\mathrm{duck}\; \end{array}\right)$	Calibrated from [13], [17]^2^
The scaling constant for farm size	θ=7490	[13]
Infectious duration for chicken farm	Gamma (mean = 1.05 + 0.36 × log($n_{j}$) + 0.06 × log($n_{j}$)^2^, variance = 0.195)	Estimate from within-farm SEIR model^3^
Infectious duration for turkey farm	Gamma (mean = 1.14 + 0.89 × log($n_{j}$) + 0.04 × log($n_{j}$)^2^, variance = 0.206)	Estimate from within-farm SEIR model^3^
Infectious duration for duck farms	Gamma (mean = 2.93 + 0.47 × log($n_{j}$), variance = 0.01 × log($n_{j}$))	Estimate from within-farm SEIR model^3^

1 The detail on the calibration of parameters is shown in Supplementary S3.

2 Modified from Hagenaars et al. (2023) [13] under the assumption that the relative susceptibility of outdoor farming is 6.3 times higher than indoor farming from Bouwstra et al. (2017) [17].

3 Estimate from a gamma distribution fitted to the infectious duration from within-farm SEIR model. The detail for within-farm SEIR model is shown in Supplementary S1 and S2.

### 2.4 Model scenario

#### 2.4.1 Baseline

The baseline scenario represents the distribution of Dutch poultry farm in 2024 (Table 1), in which 421 out of 886 layer farms (47.5%), and 46 out of 712 broiler farms operate as outdoor farms (6.5%).

#### 2.4.2 Transition from indoor to outdoor farming in layer and broiler farms

This scenario explores the transition to outdoor farming system specifically for layer and broiler farms, which are the majority of poultry farms in the Netherlands. The relative susceptibility ($\sigma_{j}$) of outdoor layer farms is assumed to be 6.3 times higher than that of indoor layer farms, and the same assumption is applied to outdoor versus indoor broiler farms [17]. Two spatial patterns to the transition to outdoor systems are explored: 1) Random pattern: Indoor layer and broiler farms are randomly selected for conversion to outdoor farms. 2) Clustered pattern: Spatial clusters of existing outdoor layer and broiler farms are identified using the K-medoids clustering method [22]. Indoor farms located closest to the cluster medoids are prioritized for conversion to outdoor farms. Both scenarios are tested at transition levels of 20%, 40%, 60%, 80% and 100% of all layer and broiler farms.

#### 2.4.3 Outdoor farm size reduction

In broiler farms, transitioning from conventional to organic systems results in a 50% reduction in maximum poultry density (from 42 kg/m^2^ to21 kg/m^2^) [8]. Similarly, in layer farms, the maximum stocking density decreases by approximately 35% when shifting from conventional (9 hens/ m^2^) to organic systems (6 hens/m^2^) [9]. Assuming the housing area remains constant, these reductions in animal density lead to a decrease in the total number of birds per farm, which decreases both the infectiousness and susceptibility of the farm (Eq. (4)). To test the effect of farm size reduction on HPAI epidemic, we simulated scenarios in which outdoor broiler farm size is reduced by 50%, and outdoor layer farm size is reduced by 35% from the baseline scenario. This assumption is based on the idea that farmers tend to maximize production as much as regulations allow.

#### 2.4.4 Decreasing the number of farms

We explored the effect of decreasing the number of farms under an extreme scenario in which 100% of layer and broiler farms have transitioned to outdoor systems, without changing the farm sizes. This scenario aims to determine the proportion of farms that needed to be removed to reduce the number of infected farms back to the level of the baseline scenario. To evaluate this, 0–90% of layer and broiler farms were randomly removed, and the resulting epidemic sizes were compared to those in the baseline scenario.

### 2.5 Sensitivity analysis

A global sensitivity analysis was performed to evaluate the model's robustness to variations in parameter values. We conducted the sensitivity analysis under two scenarios: the baseline scenario and an extreme scenario in which 100% of broiler and layer farms are outdoor. Using Latin hypercube sampling, 600 parameter sets were drawn from the uniform distributions (Table 3). For each parameter set, the average number of infected farms in 500 model iterations was recorded. Subsequently, a regression-based method was used to quantify the outcome variance explained by the parameters [23]. A quadratic regression mode was fitted between the average number of infected farms and input parameters, and the most parsimonious model with at 90% variance explained was used. The sensitivity was explained in terms of the top marginal variance (TMV) and bottom marginal variance (BMV). TMV represents the percentage variance explained by the regression model using only that parameter. BMV represents the percentage of variance that the regression model could not explain in the absence of that parameter [24].

**Table 3:** Parameter distributions for sensitivity analysis.

Parameter	Range	Reference
Transmission kernel		[13]
- $k_{0}$	min = 0.000463, max = 0.00084	
- $r_{0}$	min = 1.47, max = 3.89	
- α	min = 1.87, max = 2.63	
- θ	min = 4510, max = 11,900	
Relative susceptibility		[17] [13]
- $\sigma_{\mathrm{outdoor layer}}$	min = 4.7, max = 8.6	
- $\sigma_{\mathrm{indoor broiler}}$	min = 0.0414, max = 0.322	

## 3 Results

### 3.1 HPAI introduction from the environment into poultry farms

**Fig. 1:**
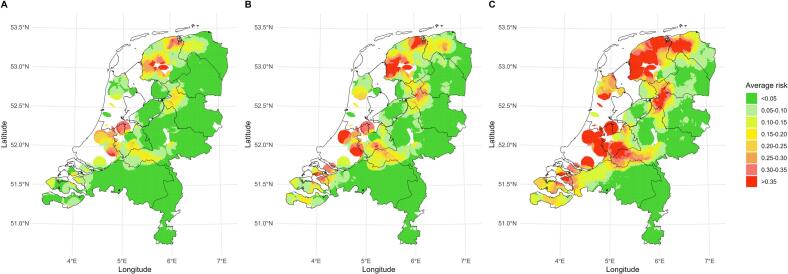
Average area-level risk of HPAI introduction from environment into poultry farms. Map A represents the scenario in which all layer and broiler farms are indoor farms. Map B represents the baseline scenario, in which 47.5% of layer farms and 6.5% of broiler farms are outdoor. Map C represents the scenario in which all layer and broiler farms are outdoor farms. The map of the Netherlands is divided into grid size of 1 km^2^, and the colour of the grid represents the average risk of farms within a 10 km radius of the grid. The risk is the probability of the farm getting infected with HPAI from the environment at least once within 9 years. The white areas represent regions where there are fewer than two farms within a 10 km radius of the square grid.

**Fig. 2:**
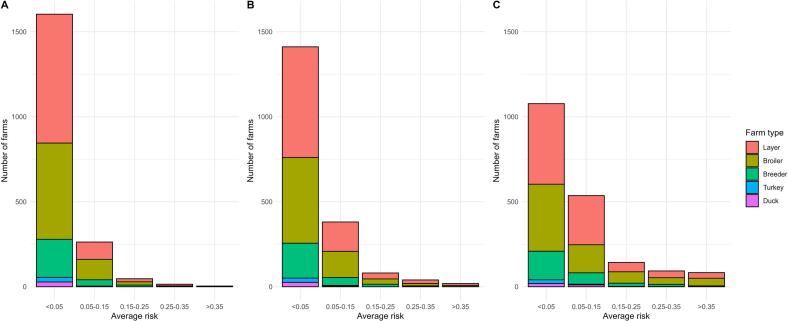
Distribution of Number of farms based on their location and estimated risk of introduction. Y-axis shows the number of farms. X-axis shows the average risk of area where the farm located, range from low (<0.05) to high (>0.35) probabilities of introduction. Graph A represents the scenario in which all layer and broiler farms are indoor farms. Graph B represents the baseline scenario, in which 47.5% of layer farms and 6.5% of broiler farms are outdoor. Graph C represents the scenario in which all layer and broiler farms are outdoor farms.

The average area-level risk of HPAI introduction from environment into poultry farms is shown in Fig. 1, based on the Dutch poultry farm 2024 data and the risk factors identified by Gonzales et al. (2022) [16]. Fig. 1 shows the maps of average area-level risk of HPAI introduction from the environment into poultry farms for the scenarios where 100% of layer and broiler farms are indoor, for the baseline scenario, and for the scenarios where 100% of layer and broiler farms are outdoor. Fig. 2 shows the number of farms located in each area-level risk. When the layer and broiler farms converted from indoor to outdoor farming systems, the area risks increased, especially in the central and northern parts of the Netherlands (Fig. 1). This resulted in more farms being located in high-risk areas (Fig. 2). The number of poultry farms in areas with an introduction probability exceeding 0.35 increased from 19 farms (i.e., 11 layers, 7 broilers and 1 breeder) in baseline scenario to 83 farms (i.e., 33 layers, 45 broiler and 5 breeders) in scenarios where 100% of layer and broiler farms were outdoor, and it decreased to 4 farms (i.e., 3 layers and 1 broiler) in scenarios where 100% of layer and broiler farms were indoor.

### 3.2 HPAI epidemic

**Fig. 3:**
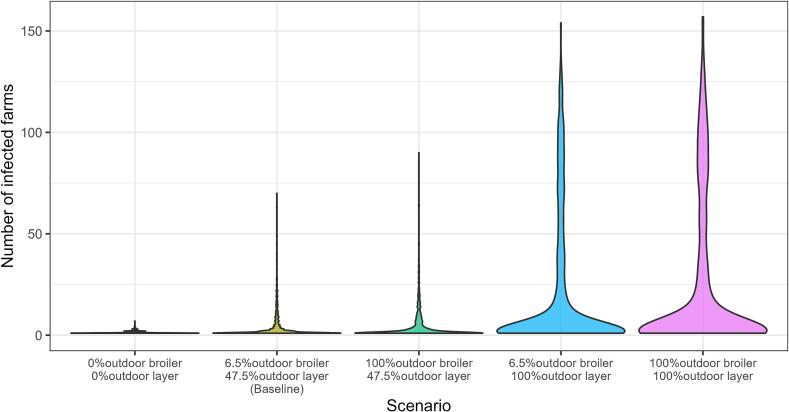
Distribution of final epidemic size from scenarios with varying percentages of outdoor broiler and layer farms. Each scenario was simulated for 1000 iterations.

The increased percentage of outdoor farms resulted in larger final epidemic size compared to the baseline (Fig. 3). Assuming 100% outdoor layer farms, the final epidemic size in the scenario with 6.5% outdoor broiler farms was barely different from that in the scenario with 100% outdoor broiler farms, reflecting that the conversion of indoor broiler farms to outdoor had less impact on increasing epidemic size compared to the conversion of indoor layer farms to outdoor. Thus, in the section below, we only show the results for scenarios where the percentage of outdoor broiler farms is kept at the baseline of 6.5%, and only the percentage of outdoor layer farms is varied. However, the results with different percentage of outdoor broiler farms were included in supplementary S6.

**Fig. 4:**
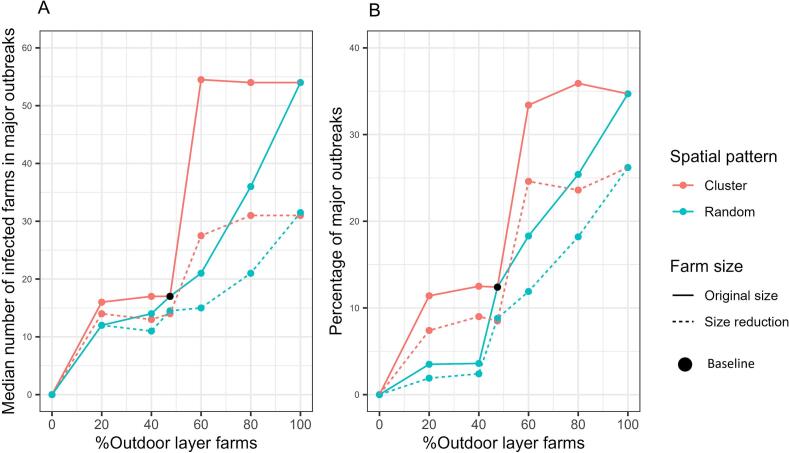
Simulated HPAI epidemics under different scenarios for layer farms while keeping 6.5% of broiler farms being outdoor (baseline scenario) fixed. Graph A shows the median number of infected farms from simulations resulting in a major epidemic (defined as ≥10 infected farms). Graph B shows the percentage of major epidemics. Colours represent the spatial distribution of outdoor farms, and line types indicate scenarios with farm size reduction. The black dots represent value from the baseline scenario.

Fig. 4 shows the median number of infected farms in major epidemics (defined as ≥10 infected farms) and the percentage of major epidemics under different scenarios, which are determined by an assumption of 6.5% of broiler farms being outdoor and varying percentages of outdoor layer farms. The spatial distribution of outdoor farms had a substantial impact on epidemic size. Epidemic sizes increased sharply in the clustered pattern, whereas increases were more gradual in the random pattern. For a same percentage of outdoor layer farms, scenarios with reduced farm sizes resulted in smaller epidemics compared to those without size reduction. The outbreak duration was not included in the manuscript but is shown in Supplementary S7.

**Fig. 5:**
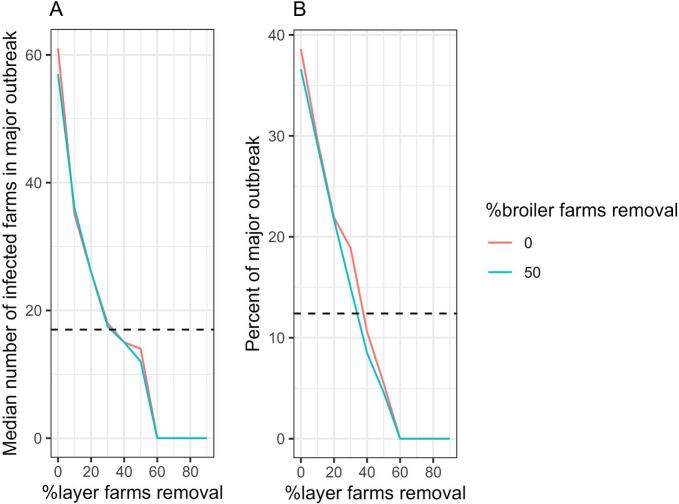
Simulated HPAI epidemics assumed 100% of broiler and layer farms were converted to outdoor systems, with varying percentages of randomly removed layer and broiler farms. Graph A shows the median number of infected farms from simulations resulting in a major epidemic (defined as ≥10 infected farms). Graph B shows the percentage of major epidemics. The x-axis represents percentage of layer farms removed, while colours indicate different percentage of broiler farm removal. Dashed line represents value from baseline scenario.

Given that 100% of broiler and layer farms were converted to outdoor systems with original farm size, reducing the number of outdoor layer farms had a greater impact on reducing epidemic size than reducing outdoor broiler farms. When approximately 35% of layer farms were removed, the median number of infected farms in major outbreak matched that of the baseline (17 infected farms). A 60% reduction in layer farms limit the epidemic to the index case, with no secondary infections (Fig. 5).

### 3.3 Sensitivity analysis

The global sensitivity analysis is shown in Table 4. In the baseline scenario, the highest TMV and BMV was $r_{0}$ followed by $k_{0}$ and $\sigma_{\mathrm{outdoor layer}}$, respectively. The $\sigma_{\mathrm{indoor broiler}}$ had the least TMV and BMV. Similar sensitivity results were observed in the 100% outdoor broiler and layer farms scenario.

**Table 4:** Global sensitivity analysis of parameters in the baseline and 100% outdoor broiler and layer farms scenarios.

Parameter	Baseline		100% outdoor broiler and layer farms	
	Top marginal variance (%)	Bottom marginal variance (%)	Top marginal variance (%)	Bottom marginal variance (%)
$k_{0}$	15.2	25.9	12.9	19.1
$r_{0}$	43.3	58.9	52.8	62.7
α	3.3	6.4	8.2	11.4
θ	3.5	11.2	2.1	4.9
$\sigma_{\mathrm{outdoor layer}}$	7.4	15.6	9.7	16.9
$\sigma_{\mathrm{indoor broiler}}$	0.3	0	–	–

## 4 Discussion

The results of this study demonstrate that the increasing number of outdoor poultry farms evidently increases the risk HPAI introduction into farms and HPAI spread between farms. More importantly, the extent of the epidemic size increase depends on the spatial pattern of outdoor farms, with a clustered pattern leading to a sharp increase in epidemic size. Reducing farm size and the number of farms decreased epidemic size, which are potential mitigation methods.

Even though several studies have shown that outdoor poultry farms increase the risk of AI introduction and spread [11], [12], [25], the quantification of this increased risk and transmission is rarely available, thus making modelling the effect of outdoor farms on the risk of HPAI challenging. Nevertheless, a study shows the relative risk of LPAI introduction in outdoor layer farms to be 6.3 times higher than that in indoor layer farms [17]. This led to our assumption to modify the susceptibility of outdoor layer and broiler farms to be 6.3 times higher than their indoor farms. With this assumption, an increased number of outdoor farms led to a higher area risk of HPAI introduction, resulting in more farms being located in high-risk areas.

Using the model, we quantified potential HPAI epidemic size under different scenarios. The result shows that outdoor layer farms greatly contributed to the increase in potential epidemic size due to the higher susceptibility of layer farms than broiler farms. The susceptibility parameters were estimated using epidemic data from 2012 to 2022 in the Netherlands [10]. Higher susceptibility in layer farms compared to broiler farms has also been reported in other studies [26]. The higher susceptibility of layer farms might be due to their longer production cycle, which increases the duration of exposure. Additionally, layer farms involve more frequent contacts with people and fomites, such as egg collection, all of which contribute to a higher risk of infection during an epidemic [27]. The spatial pattern of outdoor farms had a big impact on the increase in epidemic size. In the clustered pattern, the epidemic size increased sharply, compared to the gradual increase in the random pattern. The cluster of outdoor farms acts as a multiplier for infection, raising the probability of major epidemic and epidemic size [18]. In practice, the formation of clusters of outdoor farms is probable, as several studies have shown that the adoption of farming practices are influenced by the behaviour of neighbouring farms [28], [29], [30]. To mitigate the risk of large HPAI epidemic, the location of outdoor farms should be planned to avoid both clustering and areas with high risk or introduction.

In this study, we explore scenarios with mitigation measures, including farm size reduction and a reduced number of farms. Reducing farm size by 35% in outdoor layer farms and 50% in outdoor broiler farms, following the stocking density reduction from conventional to organic farms, reduced the epidemic size compared to scenarios with the original farm sizes. However, this effect did not fully counterbalance the overall increase in outdoor farms. In the scenario where all broiler and layer farms are outdoor, randomly removing 35% of layer farms reduced the epidemic size to that of the baseline scenario.

Other mitigation measures can be applied to reduce the risks associated with outdoor farming practices. For instance, establishing farms away from water sources and grassland can reduce the likelihood of contact between poultry and waterfowls, thereby lowering the risk of HPAI introduction. Implementing housing orders during high-risk periods to prevent direct contact between wild birds and poultry through confinement, netting, or limitation of outdoor access area of poultry [26]. Enhancing biosecurity measures on outdoor farms could also reduce the risk of HPAI introduction and transmission; for example, daily inspections, prompt removal of carcasses and eggs from outdoor areas, and preventing the formation of water pools around the facility can help mitigate these risks [31]. To enable the early detection and prompt control of HPAI outbreaks, passive surveillance based on notification of clinical signs and mortality is the most effective early detection of HPAI outbreaks in domestic poultry and wild birds [32]. In anseriform birds, where clinical signs are subtle, this should be complemented by active serological surveillance [26]. Vaccination in poultry can be considered for prevent and control the spread of HPAI [33].

Some limitations in the study should be noted. First, due to the lack of actual data from HPAI outbreaks, the susceptibility of outdoor and indoor farms was inferred from the study of LPAI. Second, our model's assumptions about farm size reduction were based on a certain percentage based on the maximum number allowed in regulations. In reality, the reduction in farm size might vary. Although we found that reducing farm size, relocating farms to prevent clustering, and reducing the total number of farms could be potential mitigations, implementing them in the real world is challenging due to economic viability, farmer compliance, and legal hurdles in zoning regulations. Further research is needed to determine if these mitigations can be translated into practical policy.

## 5 Conclusion

The rising trend of extensive poultry farms in Western Europe, particularly in the Netherlands, leads to more poultry having outdoor access. While the outdoor farming practice benefits animal welfare, our study shows that the increased number of outdoor farms increased the risk of HPAI introduction and led to larger potential epidemic sizes. To mitigate higher risk from increasing number of outdoor farms, we recommend preventing clusters of outdoor farms, reducing farm size, and/or decreasing the overall number of farms.

## CRediT authorship contribution statement

**Thanicha Chanchaidechachai:** Writing – original draft, Methodology, Formal analysis, Data curation, Conceptualization. **Arjan Stegeman:** Writing – review & editing, Funding acquisition, Conceptualization. **Francisca C. Velkers:** Writing – review & editing, Resources. **Jose L. Gonzales:** Writing – review & editing, Resources, Methodology. **Egil A.J. Fischer:** Writing – review & editing, Supervision, Project administration, Methodology, Investigation, Formal analysis, Conceptualization.

## Declaration of competing interest

None.

## Data Availability

The data that has been used is confidential.

## Appendix A Supplementary data

Model and parameter description Image 1
