# Supplementary material for: Assessing the impact of outdoor farming, farm size, and farm density on highly pathogenic avian influenza epidemics: A modelling study in the Netherlands
Source: One Health. 2026 Apr 22;22:101424. doi: 10.1016/j.onehlt.2026.101424 (PMC13141481; doi:10.1016/j.onehlt.2026.101424)
Supplement: Supplementary file 1 — Model and parameter description [file mmc1.docx]

**Supplementary file**

## S1) Within-farm transmission model

The within-farm model is modelled as a stochastic SEIR model. The parameters are specific to the animal types, i.e., chickens, turkeys and ducks (Table A1). The deterministic version of the model can be described as:

$$\frac{dS(t)}{dt}= -\beta I\left( t \right)\frac{S\left( t \right)}{N}- \mu S(t)$$

$$\frac{dE(t)}{dt}=\beta I\left( t \right)\frac{S\left( t \right)}{N}-\sigma E(t)- \mu E(t)$$

$$\frac{dI(t)}{dt}=\sigma E\left( t \right)-\gamma I(t)- \mu I(t)$$

$$\frac{dR(t)}{dt}=(1-\emptyset)\gamma I\left( t \right)- \mu R(t)$$

$$\frac{dDS(t)}{dt}= \mu S(t)$$

$$\frac{dDE(t)}{dt}= \mu E(t)$$

$$\frac{dDI(t)}{dt}=\left( \emptyset\gamma+\mu\right)I$$

$$\frac{dDR(t)}{dt}=\mu R(t)$$

Table S1.1 Parameters for within-farm HPAI model

| Parameter | Value | Reference |
| --- | --- | --- |
| Chickens  - Transmission rate  - Latent period  - Infectious period  - Mortality after infectious period | $\beta$ = 1.31 day^-1^  $1/\sigma$ = 1 day  $1/\gamma$ = 3 days  $\emptyset$= 0.99 | Germeraad et al., 2023  Hobbelen et al., 2020 Germeraad et al., 2023  Germeraad et al., 2023 |
| Turkeys  - Transmission rate  - Latent period  - Infectious period  - Mortality after infectious period | $\beta$ = 1.26 day^-1^  $1/\sigma$ = 1 day  $1/\gamma$ = 6.2 days  $\emptyset$= 0.9 | Bos et al., 2008  Hobbelen et al., 2020  Bos et al., 2008  Bos et al., 2008 |
| Ducks  - Transmission rate  - Latent period  - Infectious period  - Mortality after infectious period | $\beta$ = 4.1 day^-1^  $1/\sigma$ = 0.17 day  $1/\gamma$ = 4.3 days  $\emptyset$= 0.7 | Vergne et al., 2021 Vergne et al., 2021 Vergne et al., 2021 Vergne et al., 2021 |
| Background mortality rate | $\mu$ = 0.0005 day^−1^ | Germeraad et al., 2023 |
| Detection criteria | Mortality more than or equal to 0.5% of flock for 2 consecutive days | Backer et al., 2015 |
| Number of index cases | 10 if farm size ≥ 10;  Maximum number poultry in the farms if the farm size < 10 | Authors’ assumption |

The continuous-time Markov chain (CTMC) model is used to incorporate the stochastic process, in which stochastic discrete state transitions are determined by specific rates or probabilities.

Table S1.2 State transitions of stochastic model

| State transition | Rate |
| --- | --- |
| S 🡪 E | $\frac{\beta I}{N}$ |
| E 🡪 I | $\sigma$ |
| I 🡪 R | $(1-\emptyset)\gamma$ |
| S 🡪 DS | $\mu$ |
| E 🡪 DE | $\mu$ |
| I 🡪 DI | $\mu+ \emptyset\gamma$ |
| R 🡪 DR | $\mu$ |

We ran the stochastic within-farm SEIR models for each poultry types and farm sizes. We selected the 1^st^ to 100^th^ percentiles of farm sizes from the 2024 poultry data to run the within-farm transmission models. This approach ensures that we simulated outcomes covering the entire range of farm sizes. For each combination of farm size and poultry types, we ran the within-farm transmission models for 100 iterations. The model outcomes (i.e., infectious duration, number of sick poultry, cumulative sick animal day) were than saved and fitted with gamma distribution as explained in detail below.

## S2) Infectious duration and exposure

The simulated infectious durations from the stochastic within-farm transmission models were fitted to gamma distributions, with the mean and variance dependent to farm size. Similarly, the simulated cumulative sick-animal days were fitted to gamma distributions, with the mean and variance dependent by both farm size and infectious duration. These gamma distributions were then used to draw infectious durations and cumulative sick-animal days for the between-farm transmission model.

Table S2.1 Summary of gamma distributions fitting with the outcomes from within-farm transmission model

| Model outcomes | Distribution |
| --- | --- |
| Infectious duration for chicken farm | Gamma (mean = 1.05 + 0.36 × log(farm size) + 0.06 × log(farm size)^2^, variance = 0.195) |
| Infectious duration for turkey farm | Gamma (mean = 1.14 + 0.89 × log(farm size) + 0.04 × log(farm size)^2^, variance = 0.206) |
| Infectious duration for duck farms | Gamma (mean = 2.93 + 0.47 × log(farm size),  variance = 0.01 × log(farm size)) |
| Cumulative sick-animal days for chicken farm | Gamma (mean = 8.38 + 0.39 × farm size + 44.61 × infectious duration,  variance = 1 + 219.93 × farm size + 1.03 × infectious duration) |
| Cumulative sick-animal days for turkey farm | Gamma (mean = 1.30 + 1.86 × farm size + 69.34 × infectious duration,  variance = 1.05 + 1068.5 × farm size + 1.72 × infectious duration) |
| Cumulative sick-animal days duck farms | Gamma (mean = 2.22 + 0.48 × (farm size × infectious duration)  var = 0.74 + 91.82 × (farm size × infectious duration) |

## S3) Calibration of transmission kernel

The between-farm transmission models in this study used the transmission kernel parameters calibrated from the report of Hagenaars et al., (2023). In that report, the transmission kernel parameters estimated from the HPAI outbreak data in the Netherlands from 2003 – 2018. The original transmission kernel parameters are shown in Table A4.

Table S3.1. Transmission kernel parameters estimated from the HPAI outbreak data in the Netherlands from 2003 – 2018 by Hagenaars et al., (2023).

| Parameter | Value |
| --- | --- |
| Transmission kernel function: | $k_{0}$ = 0.00216 day^-1^  $r_{0}$ = 2.5  $\alpha$ = 2.2 |
| Relative susceptibility of susceptible farm | $\sigma_{j}= \left\{ \begin{aligned} 1, if j= layer \\ 0.134, if j= broiler \\ 0.377, if j=duck \\ 3 if j= turkey \end{aligned} \right.$ |
| The dependence of farm size | $\theta=7490$ |
| Infectious duration | 11.8 days |

However, these transmission kernel parameters did not differentiate between the relative susceptibility of indoor and outdoor systems. According to Bouwstra et al., (2017), the relative risk of HPAI introduction is 6.3 times higher for outdoor farming compared to indoor farming. Based on this, we assumed a relative susceptibility of 6.3 for outdoor layer farms and 0.8442 for outdoor broiler farms. In addition, Hagenaars et al., (2023) assumed a constant infectious duration of 11.8 days. In contrast, our model assumed infectious durations based on the stochastic within-farm model. Consequently, we need to calibrated the $k_{0}$ parameter to ensure that simulations using the new relative susceptibility values and varying infectious durations produce results consistent with those simulated using the original parameters.

To calibrate the parameter, we first calculated the reproduction number of each farms using Hagenaars et al., (2023) parameters based on Dutch poultry farm 2024 data. The reproduction number of each farm *i* (*Ri*) can be calculated by the sum of probability of infection from infected farm *i* to all susceptible farms *j* with each their own distance to farm *i* during infectious period of farm *i (Ti*) [6].

$$R_{i}= \sum_{j\neq i} (1-E\left[ e^{-k\left( r_{ij} \right)Ti} \right])$$

We then calibrated the parameter k0 by identifying the value that produced the closest reproduction number for each farm, given the changes in relative susceptibility and infectious duration. The best fitted $k_{0}$ parameter is determined to be 0.000574.

We compared the outbreaks simulating from parameters of Hagenaars et al., (2023) and newly calibrated parameter (Table 2 in manuscript). The comparison is shown in Table S3.2. The results showed that the new k0 parameter produces similar outcomes to those from outbreaks simulated using the original parameters.

Table S3.2 The outcomes of simulated outbreaks based on poultry farm 2024 data with the parameters from Hagenaars et al., (2023) and with the calibrated $k_{0}$ parameter. The simulation was run for 1000 iterations.

| Outcomes* | Outbreak simulations with the parameters from Hagenaars et al., (2023) | Outbreak simulations with $k_{0}$ parameter = 0.000574, and infectious duration based on the stochastic within-farm model |
| --- | --- | --- |
| Number of infected farms | mean = 11, median = 7 , 5% =2 , 10% = 2 , 90% = 25 , 95% = 31 | mean =11.4 , median = 7 , 5% = 5% 2 , 10% = 2 , 90% = 28 , 95% = 37 |
| Outbreak duration | mean = 36.1 , median = 30.6 , 5% =11.8 , 10% = 11.8, 90% =67.9 , 95% = 79.2 | mean = 31.2 , median = 26.4, 5% = 12 , 10% = 12.5 , 90% = 58.7 , 95% = 70.7 |
| Number of farms with Rh > 0.95 | 67 | 74 |
| %iteration with Outbreak size >10 | 38.6% | 37.1% |
| %iteration with Outbreak size >20 | 15.6% | 16.8% |
| %iteration with Outbreak size >40 | 2.1% | 3.9% |

* The index cases are two random farms from densely populated poultry areas to follow the assumption of Hagenaars et al., (2023).

## S4) Risk of introduction of HPAI from the environment into the farms

The risk of HPAI introduction into a poultry farm was estimated based on odds ratios from the poultry type, geographical location and environmental factors [7]. The risk of HPAI introduction from environment into the susceptible farm *j* is calculated as follows:

| $p_{j}= \frac{1}{1+ e^{-\left( {\beta_{0}+ \beta}_{j}X_{j} \right)}}$ | Eq 1 |
| --- | --- |

Where $\beta_{0}$ is the intercept of logistic regression, which is -3.466. The $\beta_{j}$ is the is the vector of regression coefficients for the environmental factors, which is equivalent to the log(odds ratio), and the $X_{j}$ is the vector of variables of farm *j.* The risk is interpreted as the probability of the farm getting infected with HPAI from the environment at least once within 9 years, assuming the association between variables and HPAI introduction remains the same as the period of 2014 – 2022.

Table S4.1 Odds ratios for the risk of highly pathogenic avian influenza introduction into a poultry farms estimated from outbreak data in the Netherlands in the period of 2014 - 2022

| Risk factors^1^ | Odds ratio |
| --- | --- |
| Poultry farm types  - Breeder  - Layer  - Broiler  - Turkey  - Duck | Reference  0.51  0.21  6.97  4.75 |
| Farming system^2^  - Indoor  - Outdoor | Reference  6.3 |
| Log(the area of water within a 500-meter radius from the farm (hectare)+1) | 2.25 |
| The shortest distance from the poultry farm to a water area (meter) | 0.98 |
| The area of grassland within a 1-kilometer radius from the farm (hectare) (knot1)^3^ | 7.98 |
| The area of grassland within a 1-kilometer radius from the farm (hectare) (knot2)^3^ | 5.98 |
| The shortest distance from the farm to a forest | 1.0003 |
| Log(number of inhabitants per square kilometer) | 0.77 |
| Geographical coordinate  - X  - Y | -0.00001  0.000005 |

^1^ The intercept of logistic regression is -3.466.

^2^ The odds ratio of farming system is assumed based on the relative risk of low pathogenic avian influenza introduction from Bouwstra et al. (2017).
^3^ The area of grassland is fitted with spline. The knot for the splines in the area of grassland variable is 104.36.

## S5) Poultry farm distribution

The distribution of Dutch poultry farms in 2024 is shown in the map below. Farm counts are displayed per 10 × 10 km grid cell.


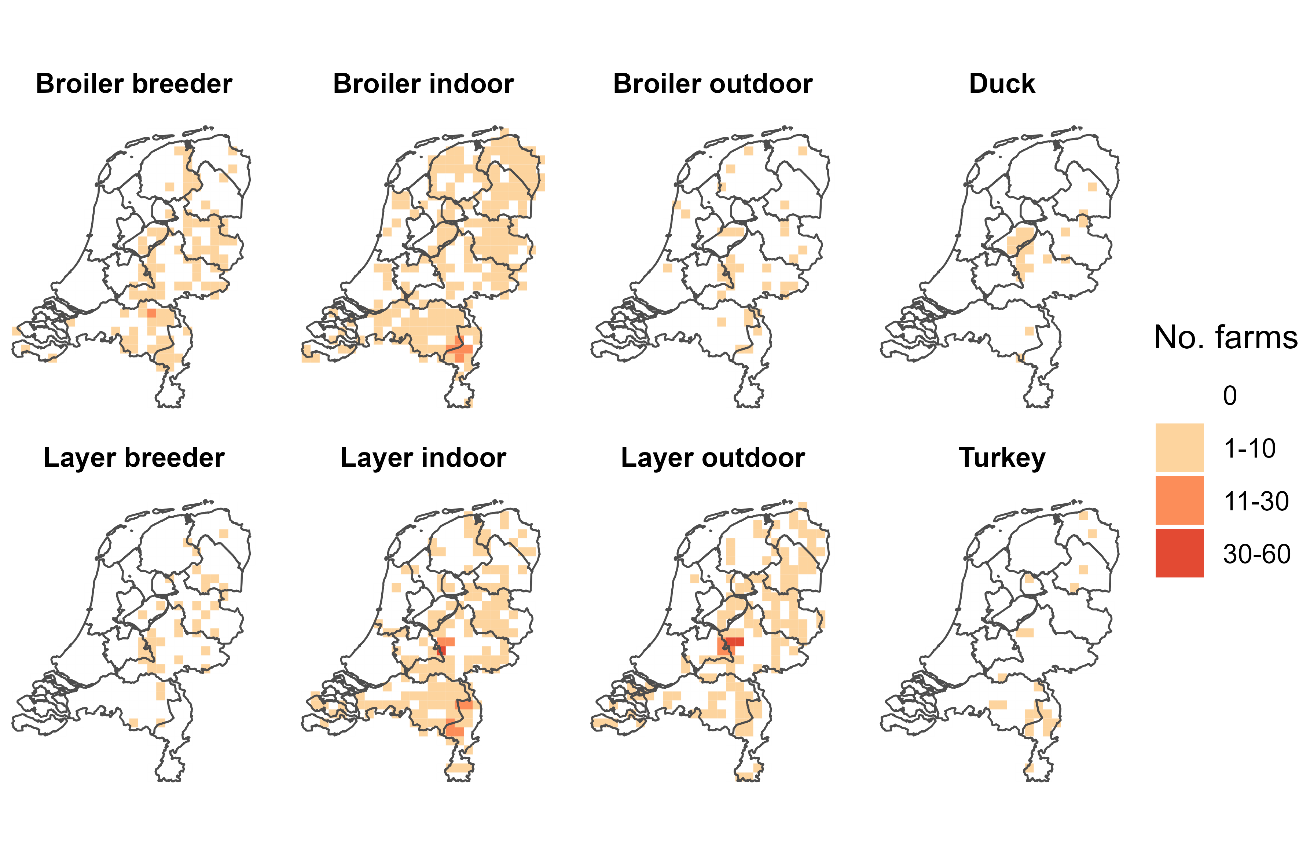


Figure S5.1 The distribution of Dutch poultry farms in 2024 is shown in the map below. Farm counts are displayed per 10 × 10 km grid cell.

## S6) The effect of outdoor broiler farms

In the manuscript, we only present the results of simulations while keeping 6.5% of broiler farms as outdoor farms (baseline scenario). The graph below shows the simulation results when the percentage of outdoor broiler farms varies from 6.5% to 100%. The results indicate that changes in the percentage of outdoor broiler farms have only a small effect on outbreak size and the percentage of major outbreaks.


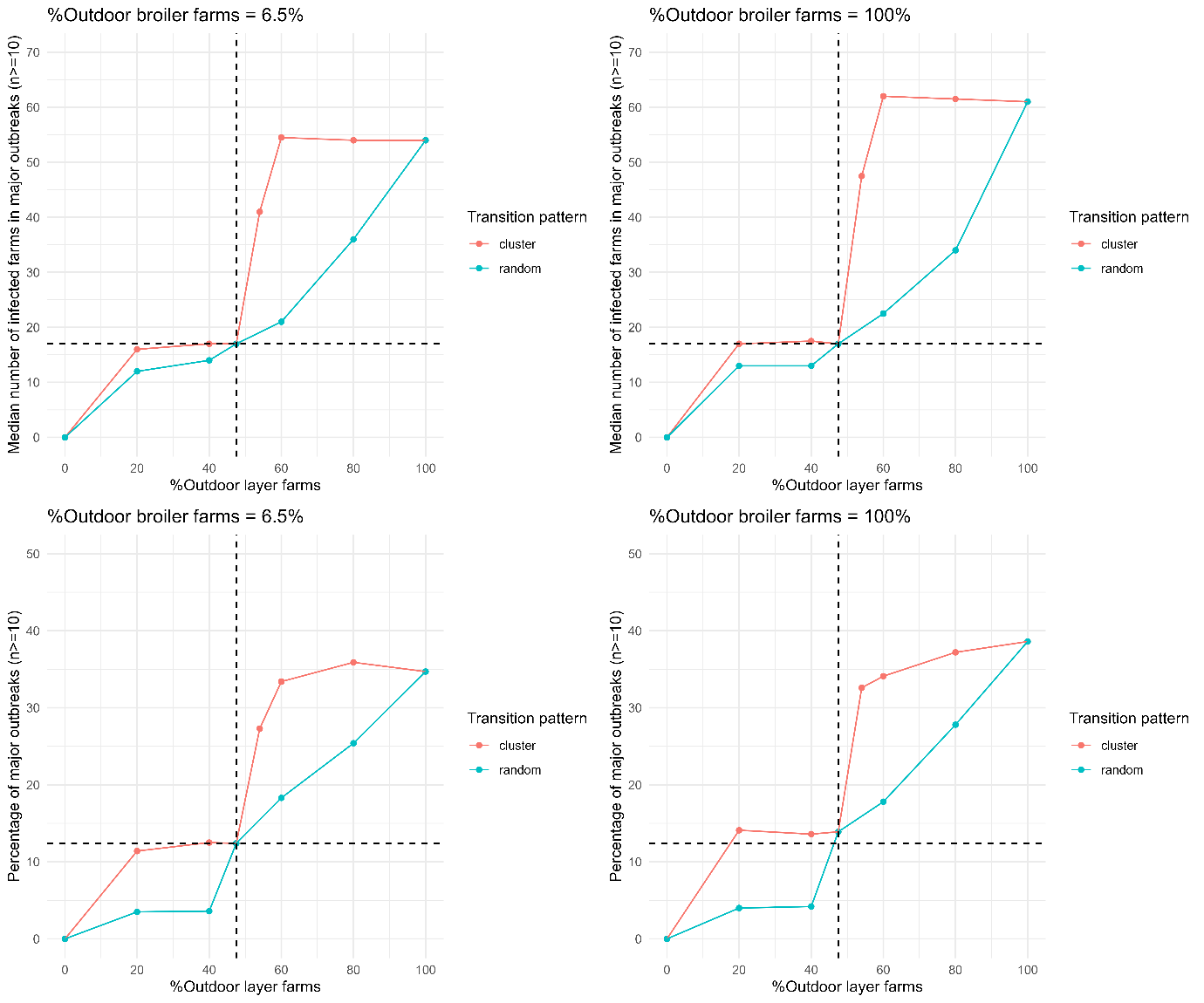


Figure S6.1 Simulated HPAI epidemics under different percentage of layer farms and broiler farms being outdoors. The upper graphs show the median number of infected farms from simulations resulting in a major epidemic (defined as ≥10 infected farms). The lower graphs show the percentage of major epidemics. The left side represents the scenario where the percentage of outdoor broiler farms is 6.5%, and the right side represents the scenario where the percentage of outdoor broiler farms is 100%. Colours represent the spatial distribution of outdoor farms. The black dots represent value from the baseline scenario.

## S7) Outbreak duration

The median outbreak duration (days) from major outbreaks (number of infected farms ≥ 10) were plotted in Figure S8.1. The median outbreak duration at baseline scenario (47.5% outdoor layer farms and 6.5% outdoor broiler farms) was 50 days. The spatial distribution of outdoor farms had a substantial impact on epidemic size. Outbreak duration increased sharply in the clustered pattern, whereas the increase was more gradual in the random pattern.


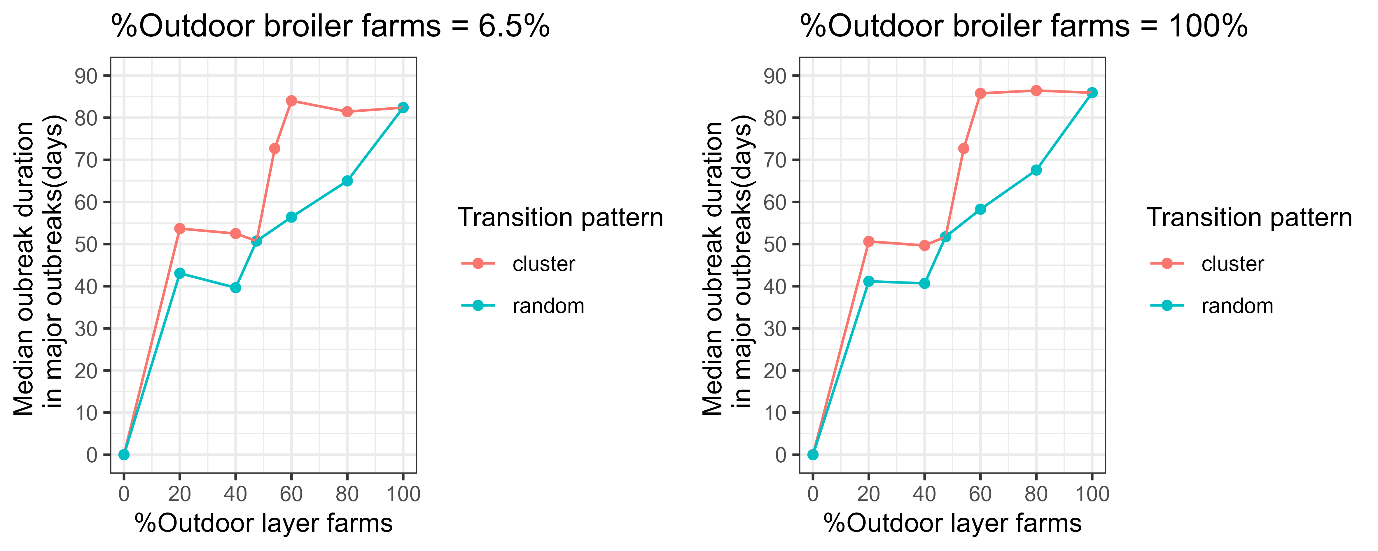


Figure S8.1 Simulated HPAI epidemics under different percentage of layer farms and broiler farms being outdoors. Y-axis show the median outbreak duration (days) in major epidemic (defined as ≥10 infected farms). The left side represents the scenario where the percentage of outdoor broiler farms is 6.5%, and the right side represents the scenario where the percentage of outdoor broiler farms is 100%. Colours represent the spatial distribution of outdoor farms.

We used the Cox proportional hazards regression model to analyse the effect of percentage of outdoor layer farms, percentage of broiler farms and spatial pattern on the outbreak duration (time-to-event). The results showed that higher percentage of outdoor layer and broiler farms is associated with a longer outbreak duration. Outbreaks in a random spatial pattern ended 31% faster than in clustered patterns.

Table S7.2 Cox proportional hazards regression model to analyse the effect of percentage of outdoor layer farms, percentage of broiler farms and spatial pattern on the outbreak duration (time-to-event).

| Variable | Coef | HR (exp coef) | SE | p-value |
| --- | --- | --- | --- | --- |
| percentage of outdoor layer farms | −0.01348 | 0.9866 | 0.000150 | < 2e-16 |
| percentage of broiler farms | −0.000726 | 0.9993 | 0.0000910 | 1.49e-15 |
| Spatial pattern: random | +0.2710 | 1.311 | 0.00816 | < 2e-16 |

## References

[1] E.A. Germeraad, F.C. Velkers, M.C.M. de Jong, J.L. Gonzales, J.J. de Wit, J.A. Stegeman, N. Beerens, Transmissiestudie met vier vaccins tegen H5N1 hoogpathogeen vogelgriepvirus (clade 2.3.4.4b), Wageningen Bioveterinary Research, Lelystad, 2023. https://doi.org/10.18174/584306.

[2] P.H.F. Hobbelen, A.R.W. Elbers, M. Werkman, G. Koch, F.C. Velkers, A. Stegeman, T.J. Hagenaars, Estimating the introduction time of highly pathogenic avian influenza into poultry flocks, Sci. Rep. 10 (2020) 12388. https://doi.org/10.1038/s41598-020-68623-w.

[3] M.E.H. Bos, M. Nielen, G. Koch, A. Stegeman, M.C.M. De Jong, Effect of H7N1 vaccination on highly pathogenic avian influenza H7N7 virus transmission in turkeys, Vaccine 26 (2008) 6322–6328. https://doi.org/10.1016/j.vaccine.2008.09.022.

[4] T. Vergne, S. Gubbins, C. Guinat, B. Bauzile, M. Delpont, D. Chakraborty, H. Gruson, B. Roche, M. Andraud, M. Paul, J. Guérin, Inferring within‐flock transmission dynamics of highly pathogenic avian influenza H5N8 virus in France, 2020, Transbound. Emerg. Dis. 68 (2021) 3151–3155. https://doi.org/10.1111/tbed.14202.

[5] J.A. Backer, H.J.W. van Roermund, E.A.J. Fischer, M.A.P.M. van Asseldonk, R.H.M. Bergevoet, Controlling highly pathogenic avian influenza outbreaks: An epidemiological and economic model analysis, Prev. Vet. Med. 121 (2015) 142–150. https://doi.org/10.1016/j.prevetmed.2015.06.006.

[6] T. Chanchaidechachai, M.C.M. de Jong, E.A.J. Fischer, Spatial model of foot-and-mouth disease outbreak in an endemic area of Thailand, Prev. Vet. Med. 195 (2021) 105468. https://doi.org/10.1016/j.prevetmed.2021.105468.

[7] J.L. Gonzales, W.H.G.J. Hennen, R. Petie, E. de Freitas Costa, N. Beerens, R. Slaterus, T. Kuiken, J. Stahl, A.R.W. Elbers, Risicofactoren voor introductie van HPAI-virus op Nederlandse commerciële pluimveebedrijven, 2014-2022, Wageningen Bioveterinary Research, Netherlands, 2022. https://edepot.wur.nl/586242 (accessed August 19, 2024).
